# The application and effect of presuturing with clips in endoscopic full-thickness resection

**DOI:** 10.1097/MD.0000000000039500

**Published:** 2024-08-30

**Authors:** Zhaohui Liu, Rui Li, Dayong Sun, Shihua Ding, Ruinuan Wu

**Affiliations:** a Department of Gastroenterology, Shenzhen Second People’s Hospital, First Affiliated Hospital of Shenzhen University Health Science Center, Shenzhen, China; b Department of Gastroenterology, First Affiliated Hospital of Soochow University, Suzhou, China; c Department of Pathology, First Affiliated Hospital of Shenzhen University Health Science Center, Shenzhen Second People’s Hospital, Shenzhen, China.

**Keywords:** antibiotics, clips, endoscopic full-thickness resection, ESR-EB, perforation, presuture

## Abstract

There are few studies on presuturing for full-thickness resection. To explore the effect of using clips as a presuturing technique for endoscopic snare resection with an elastic band (ESR-EB). The clinical data of patients who underwent ESR-EB at Shenzhen Second People’s Hospital between May 2023 and May 2024 were collected. The patients were divided into presuture and non-presuture groups according to whether tissues were stitched before resection. The general clinical characteristics, tumor growth position, tumor size, tumor growth pattern, pathological type, operation time, resection time, complication rate, number of clips, and postoperative antibiotic usage rate were compared. A total of 73 patients were enrolled, 55 of whom were included in the presuture group and 18 were included in the non-presuture group. There was no difference in age, sex, tumor position, tumor size, or tumor growth pattern between the 2 groups (*P* > .05). There was no significant difference between the 2 groups in terms of operation time, resection time, pathological diagnosis, number of clips, or complication rate (*P* > .05). Complete resection was achieved in all of the patients. The perforation diameter in the presuture group was significantly smaller than that in the non-presuture group ([3.20 ± 1.56] vs [4.67 ± 2.79], [*P* = .006]). Thirty-three (60%) patients in the presuture group and 16 (88.89%) patients in the non-presuture group received postoperative preventive antibiotics, and the difference between the 2 groups was significant (*P* = .024). Gastric myometrial lesions <10 mm in diameter can be completely removed via ESR-EB. Clips as a means of presuturing can significantly reduce the perforation diameter and the use of postoperative preventive antibiotics. Moreover, clips as a means of presuturing does not increase the total number of clips used for the procedure and therefore should be considered a feasible, safe and effective technique.

## 1. Introduction

Gastrointestinal stromal tumors (GISTs) are the most common lesions arising from the muscularis propria of the stomach.^[1,2]^ According to the WHO, the International Classification of Diseases (ICD)-10 code for GISTs is 3,^[3]^ which indicates malignancy. For small gastric muscularis propria lesions, preoperative diagnosis is difficult.^[4]^ Furthermore, according to European guidelines, endoscopic resection is less traumatic and avoids unnecessary follow-up if a histological diagnosis is not available.^[5]^

ESR-EB has recently been reported to be suitable for the resection of gastric muscularis propria lesions <10 mm in diameter.^[6,7]^ Moreover, a study of ESR-EB for the resection of gastric muscularis propria lesions revealed that ESR-EB, which is a simple and short procedure, has a 100% resection rate.^[8]^ Since resection occasionally yields large perforations that make suture closure difficult, we designed a method in which clips used to clamp the mucosa around the wound before snare resection to avoid a large wound after resection were used as a means of presuturing, which made the operation less difficult. We report the results of the study herein.

## 2. Materials and methods

### 2.1. Study design and ethics

This was a single-center retrospective study conducted at Shenzhen Second People’s Hospital. The study was conducted in accordance with the 2008 revision of the Helsinki Declaration and was approved by the Ethics Committee of the Second People’s Hospital of Shenzhen Municipality.

The clinical data of patients who underwent ESR-EB for the resection of gastric muscularis propria lesions between May 2023 and May 2024 were collected, and informed consent was obtained from each patient prior to endoscopic resection. The requirement to obtain consent to use data obtained by the institution was waived due to the retrospective nature of the study.

### 2.2. Patients

**Figure 1.**
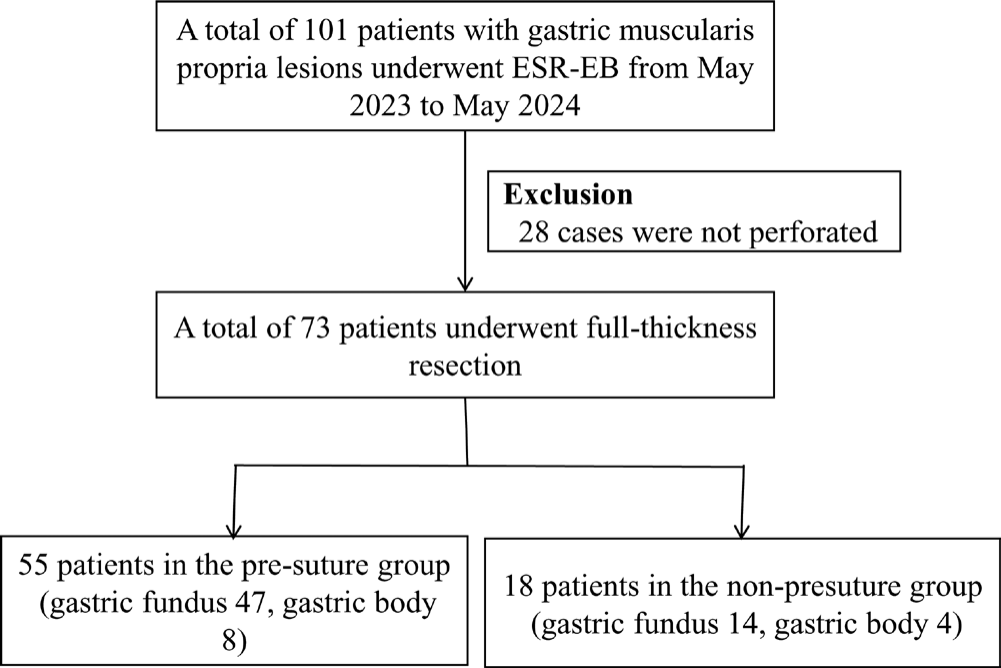
Flowchart showing patients enrolled in this study. ESR-EB: endoscopic snare resection with an elastic band.

Of 101 patients who underwent ESR-EB for the resection of gastric muscularis propria lesions, 73 underwent full-thickness resection, whereas 28 did not. Among these patients, 55 underwent placement of a presuture clip, whereas 18 did not (Fig. 1).

### 2.3. Indications for ESR-EB

ESR-EB is indicated for the following: patients with lesions originating from the gastric muscularis propria; patients with endoscopic ultrasound images showing a lesion with a diameter was <10 mm; patients with preoperative CT images showing no lymph node or distant metastasis; and patients with a generally stable condition without severe cardiopulmonary insufficiency and who can tolerate endoscopic surgery. The operator decides whether to place a presuture clip.

### 2.4. Instruments

An endoscopic image processor (Olympus, Japan, CLV-290 sl), therapeutic gastroscope (Olympus, HQ260J, Japan), high-frequency electrical generator (Elbo, VIO300D, Germany), snare (Boston Science, M00561231), hemostatic clip (Nanjing minimally invasive, China, POCC-D-26-195), and endoscopic device (Boston Science, M00542251) were used. Biopsy forceps (Anrui, China, AMHBFE2.4*1800) were used.

### 2.5. Presuture procedure

**Figure 2.**
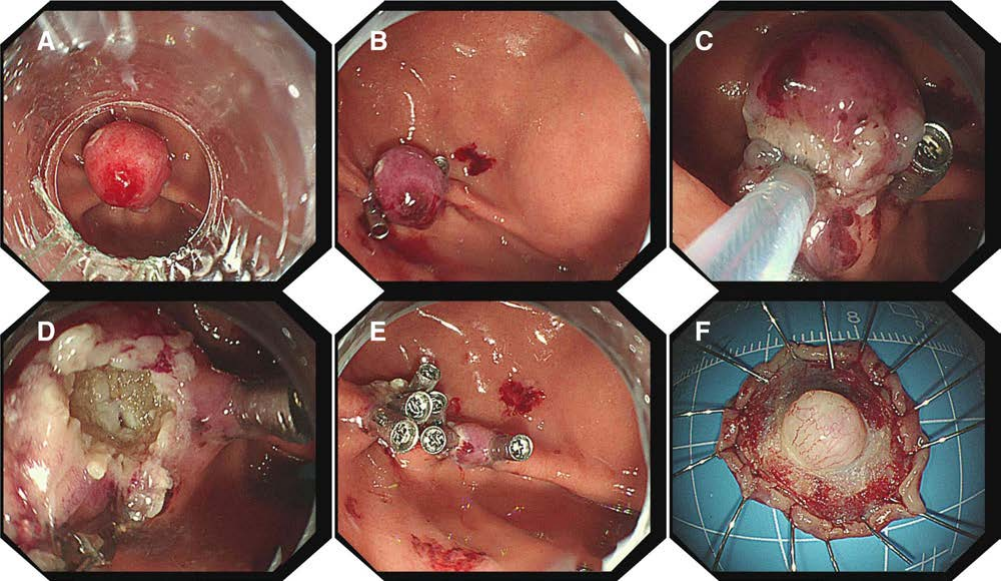
Presuturing procedure. (A) The tumor was found during white-light endoscopy. The tumor was ligated with elastic band. (B) The presuture clip was placed next to the ligated lesion. (C) The snare was placed under the elastic band for excision. (D) The wound surface was observed. (E) The wound was closed with clips. (F) The specimen was sent for pathological examination.

The ligation devices were installed on the front end of the endoscope. The gastroscope was inserted into the gastric cavity to visualize the lesions. The lesion was then pulled into the transparent cap, and the elastic band was released for ligation (Fig. 2A).The presuture clip was placed next to the ligated lesion, where the gastric mucosa appeared more wrinkled (Fig. 2B).The snare was placed under the elastic band, and the electric generator was used for slow electric cut (Fig. 2C). The surface of the wound was observed (Fig. 2D). The wound was closed with clips (Fig. 2E).The specimen was sent for pathological examination (Fig. 2F).

### 2.6. Non-presuture procedure

In the non-presuture group, the snare was placed directly under the rubber band, and the electrical generator was connected for slow electric cut. The other steps were the same as those for the presuture procedure.

### 2.7. Specimen processing

All specimens were processed and fixed in 10% formalin solution for 6 to 24 hours. The sections were then stained with hematoxylin and eosin, and all specimens were subjected to immunohistochemistry using antibodies against CD117, DOG 1, CD34, SDHB, and Ki67. Pathological diagnosis was made by a gastrointestinal pathologist in our hospital who evaluated the tumor size, risk grade, resection margin, and vascular invasion.

### 2.8. Clinical outcomes

We analyzed the complete resection rate, operation time, resection time, complication rate, antibiotic usage rate, and number of clips. Complete resection was defined as no macroscopic mass remaining after endoscopic tumor resection and negative resection margins according to the histological examination. The operation time was defined as the time from gastroscope insertion to gastroscope removal from the oral cavity. The resection time was defined as the time from when the transparent cap made contact with the lesion to the completion of wound suturing. Intraoperative bleeding was defined as a bleeding volume >10 mL. Intraoperative perforation was defined as the visualization of intra-abdominal organ tissue or omentum. The size of the perforation was defined as the maximum diameter of the breach and was measured using biopsy forceps. The use of antibiotics was determined by the physician personal experience, and there was no uniform criteria for reference.

### 2.9. Statistical analysis

All data were statistically analyzed using SPSS28 statistical software. Count data (e.g. sex, tumor growth pattern, lesion location, histological diagnosis, antibiotics, and complication rate) were analyzed by using χ^2^ tests, and expressed by n (%).^[9]^ Continuous variables (such as age, tumor size, operation time, resection time, perforation size, and number of clips) were analyzed by using variance analysis. *P* value < .05 was considered to indicate statistical significance.

## 3. Results

### 3.1. Baseline characteristics of the 2 groups

A total of 73 patients were enrolled, including 55 patients who underwent the presuturing technique, and 18 who did not. There was no difference in age, sex, tumor location, tumor size, or growth pattern (*P* > .05), so it was not necessary to match the 2 groups (Table 1).

**Table 1:** Comparison of the baseline clinical characteristics between the two groups.

	Presuture, n = 55	Non-presuture, n = 18	*F*/χ^2^	*P* value
Age (yr)	57.15 ± 9.26	56.00 ± 9.94	0.200	.656
Sex, n (%)			0.229	.633
Male	18(32.73)	7(38.89)		
Female	37(67.27)	11(61.11)		
Location, n (%)			0.582	.446
Body	8(14.55)	4(22.22)		
Fundus	47(85.45)	14(77.78)		
Tumour size (mm)	6.02 ± 1.40	5.66 ± 1.31	0.924	.656
Growth pattern, n (%)			4.170	.244
Intraluminal growth	47(85.45)	15(83.33)		
Extraluminal growth	6(10.91)	3(16.67)		
Mixed growth	2(3.64)	0(94.29)		

### 3.2. Comparison of treatment results between the 2 groups

There were no significant differences in operation time, resection time, histological diagnosis, complication rate, and number of clips between the 2 groups (*P* > .05). Complete resection was achieved in all of the patients. The perforation diameter in the presuture group was significantly less than that in the non-presuture group ([3.20 ± 1.56] mm vs [4.67 ± 2.79] mm), and there was a significant difference between the 2 groups (*P* = .006). The postoperative utilization rate of antibiotics was 60.00% in the presuture group and 88.89% in the non-presuture group, and there were significant differences between the 2 groups (*P* = .024; Table 2).

**Table 2:** Comparison of treatment results between the two groups.

	Presuture, n = 55	Non-presuture, n = 18	*F*/χ^2^	*P* value
Operation time (min)	20.09 ± 8.20	18.78 ± 5.12	0.408	.525
Resection time (min)	8.96 ± 3.53	9.56 ± 2.91	0.412	.523
Complete resection, n (%)	55(100.00)	18(100.00)		
Histology diagnosis			0.265	.607
Leiomyoma, n (%)	4(7.27)	2(11.11)		
GISTs, n (%)	51(92.73)	16(88.89)		
Perforation size (mm)	3.20 ± 1.56	4.67 ± 2.79	7.881	.006**
Intraoperative adverse events			6.561	.087
Bleeding, n (%)	0(0.00)	1(5.56)		
The tumor fell into the abdominal cavity, n (%)	0(0.00)	1(5.56)		
Electrocoagulation injury, n (%)	1(1.82)	0(0.00)		
Postoperative adverse events			1.024	.599
Fever, n (%)	1(1.82)	0(20.00)		
Abdominal pain, n (%)	1(1.82)	1(5.56)		
Antibiotics	33(60.00)	16(88.89)	5.129	.024*
Number of clips	3.96 ± 0.77	4.39 ± 0.98	3.612	.061

*P* values were calculated using the χ^2^ test for categorical data and the *t* test for continuous data.

GISTs = Gastrointestinal stromal tumors.

*P < .05; **P < .01.

## 4. Discussion

Perforation is the most common complication of endoscopic resection of gastric muscularis propria lesions. One study showed that in 69 patients, the gastric muscularis propria was removed via ESE, 23 of whom experienced perforation.^[10]^ Similarly, in another study, ESE was performed to remove gastric muscularis propria lesions in 65 patients; 8 patients experienced perforation, and the probability of perforation of the gastric fundus was 50%, which was much greater than that of the cardia and gastric body.^[11]^ Pinghong et al^[12]^ reported that of 20 gastric muscularis propria lesions excised via ESD, 3 perforations were observed, and all the lesions were located in the fundus of the stomach. In this study, we found that the perforation rate was 72.28%. Furthermore, 61 (60.40%) patients had perforation of the fundus, and 12 (11.88%) had perforation of the gastric body. It should be emphasized that all the patients in this study were successfully sutured via an endoscopic method, and none of them needed to be transferred to surgery, which may have also be related to the small lesions included in this study.

Various endoscopic suture techniques, including the basic titanium stitching technique, the titanium clamp-nylon rope suture technique, the 3-arm stitching,^[13]^ and the omental suture technique^[14]^ have been developed. Despite the feasibility of these techniques for most perforations, some centers are unequipped to allow their implementation. In some cases, endoscopic suturing cannot be completed, so conversion to surgical suturing is required. Conversion increases medical burden and surgical trauma. Jeong et al^[15]^ performed ESD on 27 gastric muscularis propria lesions, and perforation occurred in 2 patients. The general condition of 1 patient improved after conservative treatment, and the other underwent additional surgery. In another study, ESE was performed to remove 69 lesions, 23 of which were perforated. Among these patients, 19 underwent clip suturing, and 4 underwent surgical suturing.^[10]^ Current research focuses on developing multiple techniques for perforation repair. There are few measures and reports on how to prevent large perforations before resection. This may help to improve the rate of endoscopic perforation and reduce the rate of surgical transfer.

Zhou et al^[16]^ secured the nylon rope around the lesion with a titanium clip in advance. When the lesion was fully removed, the nylon rope was tightened immediately to completely close the perforation. This method shortens the suturing time and reduces the amount of gastrointestinal fluid entering the abdominal cavity. However, the technique is disadvantageous in that the nylon rope and titanium clips are wrapped around the tumor in advance, thus affecting the surgical procedure, and the incision made after the suture is placed is oriented towards the abdominal cavity. Moreover, in the case of delayed bleeding after incision, endoscopy is no longer an option, so laparoscopic or open surgery is required to achieve hemostasis. The presuture method proposed in this study involves the advance placement of the clip in an area approximately 1 to 5 mm adjacent to the lesion after ligation, which is equivalent to reducing the diameter of the perforation in advance. The objective is to preferentially place the clips on the more wrinkled side to prevent large perforations after wrinkle expansion.

According to several studies, the rate of resection of gastric muscularis propria lesions <10 mm in diameter with ESR-EB is 100%.^[17]^ Pan and Shi^[18]^ removed 42 lesions of the gastric fundus muscularis propria via ESR-EB, 10 of which left perforations. In some studies, researchers have questioned whether the occurrence of perforations are related to both the location and growth pattern of the lesion.^[10]^ The rate of perforation of the gastric fundus is higher than that of the gastric body because the wall of the gastric fundus is thin, which often leads to the need for full-layer ligation. The size of perforations due to ESR-EB has yet to be studied. Our study revealed that the perforation diameter was significantly greater in the non-presuture group (4.67 ± 2.79 mm) than in the presuture group (3.96 ± 0.77 mm). In the non-presuture group, one resected lesion fell into the abdominal cavity due to a large perforation. Although the lesion that fell into the abdominal cavity was ultimately found and removed from the body under gastroscopy, the risk of abdominal tumor implantation increased. Placing a clip in advance not only reduces the size of the perforation but also eliminates the risk of the lesion falling into the abdominal cavity.

Advance placement of a clip did not increase the total number of clips used. The lack of significant differences between the 2 groups may be due to the use of advance placement of clips, which limited the size of the perforation and the number of clips used to close the wound. Li et al^[19]^ showed that routine antibiotics are not required after ESE for gastric muscularis propria lesions. However, for patients who undergo endoscopic full-thickness resection, antibiotics are recommended to prevent infection.^[20]^ In this study, full-thickness resection was performed in 73 patients, 2 of whom experienced postoperative abdominal pain and no signs of peritonitis. One patient developed chills and fever, which improved after anti-infection treatment. The number of patients who used antibiotics to prevent infection was significantly higher in the non-presuture group (16 [88.89%]) than in the presuture group (33 [60.00%]). This may have been due to the larger perforations in the non-presuture group and because doctors preferred to prescribe antibiotics to patients with larger perforations. Thus, the antibiotic use rates increased. No infection-related symptoms occurred in the 24 patients who did not use antibiotics.

Our study has several limitations. As this was a retrospective study, the size of the sample of patients was small, and randomization was not appropriate. Second, in this study, 3 endoscopists performed endoscopic resection. We were unable to avoid differences in our staff’s ability to perform endoscopic assessment and treatment, but all the endoscopists had more than 5 years of endoscopy experience and had performed more than 100 ESR-EB procedures. Third, as this was a retrospective study, each endoscopist based their decision to use a clip as a means of presuturing on their personal experience, and the number of clips used may have led to selection bias. Fourth, currently, clips are only used before suturing in ESR-EB, and whether they are suitable for other endoscopic procedures should be further investigated.

In conclusion, resection of lesions originating from the gastric muscularis propria using ESR-EB is effective and safe. For lesions with a potential risk of perforation, the size of the perforation can be significantly reduced by presuturing. Presuturing does not increase the number of clips and nearly eliminates the need for antibiotics. This presuturing technique is feasible, safe, and effective.

## Author contributions

**Conceptualization:** Zhaohui Liu, Dayong Sun, Ruinuan Wu.

**Data curation:** Zhaohui Liu, Rui Li, Ruinuan Wu.

**Formal analysis:** Zhaohui Liu, Ruinuan Wu.

**Funding acquisition:** Zhaohui Liu.

**Investigation:** Zhaohui Liu, Dayong Sun.

**Methodology:** Zhaohui Liu, Rui Li, Ruinuan Wu.

**Project administration:** Zhaohui Liu, Ruinuan Wu.

**Resources:** Shihua Ding, Ruinuan Wu.

**Software:** Shihua Ding, Ruinuan Wu.

**Supervision:** Shihua Ding, Ruinuan Wu.

**Validation:** Shihua Ding.

**Visualization:** Ruinuan Wu.

**Writing – original draft:** Zhaohui Liu, Ruinuan Wu.
